# Association of Cannabinoid CB2 Receptor Q63R Variant With Rheumatoid Arthritis in an Iranian Cohort

**DOI:** 10.1155/ijog/6182868

**Published:** 2025-06-16

**Authors:** Ali Nateghi, Samin Zamani, Alireza Tahamtan

**Affiliations:** ^1^Department of Microbiology, Golestan University of Medical Sciences, Gorgan, Iran; ^2^Golestan Rheumatology Research Center, Golestan University of Medical Sciences, Gorgan, Iran

**Keywords:** cannabinoid receptor Type 2, *CNR2*, Iran, Q63R, rheumatoid arthritis, single-nucleotide polymorphism

## Abstract

**Background:** Rheumatoid arthritis (RA) is a chronic autoimmune disease primarily affecting the joints. The endocannabinoid system plays a crucial role in maintaining immune balance by regulating immune functions. Variations in the CB2 receptor gene (*CNR2*) can disrupt intracellular signaling, impairing the regulatory functions of endocannabinoids. This dysfunction is associated with an imbalanced immune response and an increased risk of autoimmune inflammatory disorders. This study investigates, for the first time in an Iranian population, the association between the Q63R polymorphism in the *CNR2* gene and RA.

**Methods:** A total of 120 RA patients and 120 healthy controls were genotyped using the TaqMan assay. Demographic and clinical data, including gender, age, and ethnicity, were collected through questionnaires. The codominant, dominant, recessive, overdominant, and additive inheritance models were analyzed using SNPStats software.

**Results:** Logistic regression analysis revealed significant associations under the codominant, dominant, and additive inheritance models, with RR genotype carriers exhibiting more than a 2.5-fold increased risk of developing RA.

**Conclusion:** The findings of this study suggest a potential role of the *CNR2* gene in RA susceptibility among Iranian patients. However, further large-scale studies are required to better understand the contribution of the CB2 receptor to disease susceptibility and its potential clinical applications as a biomarker for diagnosis and therapeutic interventions.

## 1. Introduction

Rheumatoid arthritis (RA) is a chronic autoimmune inflammatory disease of unknown etiology that primarily affects the joints [1]. It leads to persistent synovitis, systemic complications, and an increased risk of early mortality [2]. Over time, RA causes progressive joint destruction due to synovial inflammation and tissue overgrowth, resulting in increasing disability. This process is accompanied by elevated levels of autoantibodies such as rheumatoid factor (RF) and anticyclic citrullinated peptide (anti-CCP) [2, 3]. RA affects approximately 1% of adults worldwide, with women being two to three times more likely to develop the condition than men [4]. This gender disparity suggests the involvement of hormonal and genetic factors in disease susceptibility. In addition to genetic influences, autoimmunity and environmental factors may also contribute to RA pathogenesis, though the precise mechanisms remain incompletely understood [5].

In Iran, RA affects approximately 0.37% of the population, with an economic burden exceeding $3.5 billion, based on purchasing power parity (PPP) in 2019 [6, 7]. Research indicates that genetic factors account for roughly 50% of the risk of developing RA [8]. Among these genetic risk factors, tyrosine phosphatase nonreceptor Type 22 (PTPN22) risk alleles [9], human leukocyte antigen D-related (HLA-DR) alleles [10], tumor necrosis factor–receptor associated factor 1 (TRAF1) and complement Component 5 (C5) [11], and interferon regulatory factor 5 (IRF-5) play crucial roles in RA susceptibility. To date, HLA-DRB1 remains the most significant known genetic risk factor for RA [12, 13].

The endocannabinoid system (ECS) is a complex signaling network consisting of receptors, endogenous cannabinoids, and enzymes that regulate various physiological processes, including pain, mood, appetite, and immune responses [14]. Dysfunction in the ECS has been linked to several diseases, and its modulation is being explored as a potential therapeutic approach for neurodegenerative, inflammatory, and metabolic disorders, as well as cancer. Additionally, research indicates that genetic variations within the ECS can impact immune function, influencing disease severity and progression [15, 16]. Two primary cannabinoid receptors, CB1 and CB2, belong to the G-protein-coupled receptor (GPCR) superfamily [17]. CB1 is predominantly expressed in the central nervous system, where it plays a crucial role in regulating pain, mood, and appetite. In contrast, CB2 is primarily found in the peripheral immune system, where it is involved in inflammation control and immune regulation [17, 18].

The CB2 receptor is encoded by the cannabinoid CB2 receptor gene (*CNR2*), located on chromosome 1p36.11 [19]. A missense mutation in this gene results in a glutamine-to-arginine substitution at position 63 (Q63R polymorphism), altering receptor polarization and function [20, 21]. This Q63R polymorphism has been linked to several autoimmune diseases, including celiac disease (CD) [19], childhood immune thrombocytopenic purpura (ITP) [22], oligo/polyarticular juvenile idiopathic arthritis (JIA) [23], and multiple sclerosis (MS) [24]. Additionally, its association with RA was previously investigated in a Lebanese population [13]. The present study is aimed at examining, for the first time in an Iranian population, the potential role of the CB2 Q63R variant in genetic susceptibility to RA.

## 2. Materials and Methods

### 2.1. Subjects

The patient group included 120 individuals diagnosed with RA (97 women and 23 men) with a mean age of 50.1 years. The inclusion criterion required a diagnosis of RA according to the 2010 ACR/EULAR classification criteria (American College of Rheumatology standards), while the exclusion criterion was the presence of any other disease associated with CB2 Q63R polymorphisms. The control group consisted of 120 healthy individuals (100 women and 20 men) with a mean age of 50.3 years. Participants in this group were required to be free of RA and matched to RA patients in age and gender, while those diagnosed with any immune-related or chronic inflammatory diseases were excluded. All study subjects were from Golestan Province, Gorgan, Iran, and none were related. Each participant was informed about the study's purpose, provided written informed consent, and completed a demographic questionnaire. This study was approved by the Science and Bioethics Committee of Golestan University of Medical Sciences (IR.GOUMS.REC.1397.328).

### 2.2. Genotyping

Extraction of the genomic DNA was performed using a commercial kit from 300 *μ*L of whole blood samples following manufacturer's instructions (FavorPrep Blood Genomic DNA Extraction Mini Kit, Taiwan). Genotyping of all samples for the single-nucleotide polymorphism (SNP) *CNR2* rs35761398 (Q63R) variant was performed using a TaqMan assay with commercial primers and probes (Thermo Fisher, United States): 5⁣′CTGTGAAGGTCATAGTCACGCT3⁣′ (Forward primer), 5⁣′CTCTTCTGGGCCTGCTAAGTG3⁣′ (Reverse primer), and CAGGTATGAGGG CTTCCGGCGGAG [CC/TT] GGTGGGAGGACAGGATCAGATAGA [VIC/FAM] (Probes). The genotyping step of the experiment was performed on an ABI StepOnePlus Real-Time PCR system (Applied Biosystems, United States). The Real-Time PCR was carried out under the following conditions: 94°C for 4 min followed by 35 cycles of 94°C for 50 s, 55°C for 45 s, and 72°C for 50 s.

### 2.3. Statistics

Demographic and clinical data were analyzed using SPSS 26 software (IBM, Chicago, Illinois). Allele and genotype frequencies, as well as analyses of various inheritance models—including codominant, dominant, recessive, overdominant, and log-additive—were conducted using SNPStats software (a web tool for association study analysis: https://www.snpstats.net/start.htm) [25]. Odds ratios (ORs) were reported in both crude and adjusted forms using the logistic regression model, with 95% confidence intervals (CIs) calculated. Statistical significance was defined as *p* values < 0.05. The Hardy–Weinberg equilibrium (HWE) was assessed for each SNP, and test power was calculated using G-Power 3.1.9.7 software (Universitat Kiel, Germany).

## 3. Results

An overview of the subjects' demographic, clinical, and treatment information is provided in Table 1. A total of 240 subjects were included, with 120 patients diagnosed with RA and 120 healthy controls. Among all subjects, 197 (82.1%) were female and 43 (17.9%) were male. The male-to-female ratios of the patients and controls were 1:4.2 and 1:5, respectively, with no significant difference between the two groups. The mean age of patients was 50.1 ± 13.8 years, while the mean age of controls was 50.3 ± 11.4 years. Among all participants, 33.75% were houseworkers, 11.25% were retired, 8.75% were self-employed, and 27.91% worked in other types of jobs. The positive results of RF and anti-CCP tests were reported in 78/120 (65%) and 68/120 (56.7%) of RA cases, respectively. Among patients, 56/120 (46.6%) received disease-modifying antirheumatic drugs (DMARDs), 52/120 (43.3%) received steroids, 12/120 (10%) used nonsteroidal anti-inflammatory drugs (NSAIDs), 9/120 (7.5%) were treated with azathioprine, and 2/120 (1.6%) received adalimumab.

The allele and genotype frequencies of the Q63R polymorphism are summarized in Table 2. Polymorphism frequencies were consistent with HWE in all subjects, patients, and controls (*p* > 0.05). The R allele (57.5%) was more frequent than the Q (42.5%) allele in all participants, and patients had a higher frequency of the R allele (63%) compared to controls (52%). The QR genotype was the most frequent in both patients (51%) and controls (59%). No significant association was identified between CB2 Q63R variants and the demographic or clinical characteristics of the subjects (Table 3).

Logistic regression analysis of the SNP revealed significant associations in the codominant (OR: 2.62, 95% CI: 1.15–5.96, *p* = 0.029), dominant (OR: 2.07, 95% CI: 1.17–3.64, *p* = 0.011), and additive models (OR: 1.68, 95% CI: 1.13–2.50, *p* = 0.0099). The dominant and additive models were considered the best inheritance models based on their smaller Akaike information criterion (AIC) values. Similar results were observed after adjusting for gender and age (Table 4). It is important to note that the power of the test was 0.96121.

## 4. Discussion

Although the exact cause of RA remains unknown, both genetic and environmental factors are known to contribute to its development [26]. The generation of autoreactive B and T lymphocytes in RA may result from the patient's genetic predisposition. Additionally, bacterial or viral infections and tissue injury can activate antigen-presenting cells (APCs), which, in turn, stimulate autoreactive lymphocytes. This process can lead to the loss of tissue or organ tolerance, ultimately resulting in tissue and organ destruction [27].

The ECS plays a critical role in immune homeostasis, regulating immune responses and influencing the development of immune-related diseases [28, 29]. Immune cells, particularly lymphocytes, express high levels of CB2 receptors, highlighting their involvement in immune regulation [29]. Mutations occurring in different regions of the CB2 receptor can result in various SNPs [30]. While SNPs commonly contribute to phenotypic variability between individuals and populations without necessarily causing disease, they can also influence disease pathogenesis, severity, and treatment response [31]. The rationale for this study stems from the immunopathology of RA, along with existing data on the regulatory role of CB2 in immune function.

Research suggests that the CB2 Q63R polymorphism may impact receptor signaling [30]. Additionally, studies have reported that the 63R variant exhibits impaired functionality in modulating immune responses compared to the 63Q variant [32]. T lymphocytes from individuals carrying the 63R variant demonstrate a twofold reduction in the inhibition of proliferation triggered by endocannabinoids compared to those with the 63Q variant, indicating diminished receptor functionality [20]. While this variant is unlikely to directly cause disease, it may contribute to variability in CB2-mediated functions, potentially influencing disease risk, progression, and drug response [30].

A significant difference in Q63R allele and genotype distributions was observed between RA patients and healthy controls (Table 2). The codominant, dominant, and additive inheritance models demonstrated a significant association between Q63R polymorphism and RA (Table 4). According to the codominant model, individuals with the RR genotype exhibited a more than 2.5-fold increased risk of developing RA compared to those with the QQ genotype. Similarly, under the dominant model, RR carriers had more than twice the risk of developing RA compared to QR-QQ subjects.

The association of the Q63R polymorphism with various autoimmune diseases, including MS [24], CD [19], ITP [22], JIA [23], and IBD [33], has been demonstrated in previous studies. Strisciuglio et al. reported the involvement of the Q63R variant in the pathogenesis and clinical features of pediatric IBD in Italy [33]. In contrast, a study by Yonal et al. found no association between Q63R variants and IBD in the Turkish population [34]. Additionally, Tahamtan et al. found that individuals with the RR genotype had more than a threefold increased risk of developing MS compared to those with the QQ genotype [24].

Beyond autoimmune disorders, the Q63R polymorphism has also been implicated in viral infections. In a study on Italian hepatitis C virus (HCV)–infected patients, Sagnelli et al. reported that individuals with the RR genotype had an approximately two-fold increased risk of coinfection with human immunodeficiency virus (HIV) [35]. Additionally, research by Rastegar et al. found that in coronavirus disease 2019 (COVID-19) patients, the CB2 63R variant was associated with increased disease severity in an Iranian population [36].

Among RA patients, only one study by Ismail and Khawaja reported an association between the Q63R polymorphism and RA in Lebanese patients, a finding consistent with our study [13]. Their research demonstrated that the CB2 RR genotype was significantly overrepresented in RA patients compared to healthy controls, with QR and RR genotypes conferring a three-fold and 10-fold increased risk, respectively, for developing RA compared to QQ carriers [13]. Compared to our findings, Ismail and Khawaja reported a stronger association, which could be attributed to ethnic differences between participants in the two studies. Furthermore, our study found no significant association between demographic and clinical characteristics and CB2 Q63R genotype distribution (Table 3), a result consistent with the findings of Ismail and Khawaja [13].

As previously mentioned, B and T cells play a crucial role in the pathogenesis of RA. B cells secrete RFs, anti-CCP, and proinflammatory cytokines, all of which contribute to disease progression. Additionally, T cells activate macrophages and fibroblasts, transforming them into tissue-destructive cells that damage joints in RA [37]. Since the CB2 receptor is highly expressed in lymphocytes, individuals with the RR variant may have more autoreactive T cells, potentially playing a critical role in disease initiation [24]. Given the findings of this study, it is reasonable to conclude that the CB2-R63 variant, which is associated with reduced immunomodulatory function and autoimmune diseases, is predominantly linked to autoimmune conditions like RA.

Several limitations should be considered in this study. First, genetic associations, such as the one observed between Q63R polymorphism and RA, can vary across different ethnic groups, likely reflecting the unique genetic background and environmental factors of each population. Since this study focused exclusively on Iranian participants, the findings may not be generalizable to other populations. Further research involving ethnically diverse cohorts is necessary to better understand the broader relevance of this association. Second, the study was based on a small sample size and employed only the TaqMan assay. While TaqMan genotyping offers high sensitivity, specificity, and efficiency in detecting known SNPs, it is limited by its inability to detect novel variants and the potential for genotyping errors. To strengthen and expand these findings, further studies using whole-genome sequencing with larger cohorts are needed. Third, ethnic background data for participants were unavailable. Access to this information could have provided a deeper understanding of genetic influences and improved the interpretation of study results. Finally, the study lacked data on environmental factors, such as smoking habits, and laboratory parameters, including blood cell counts. As a result, interactions between these variables and the CB2 Q63R polymorphism could not be assessed.

## 5. Conclusion

In conclusion, this study presents the first report of an association between the CB2 receptor and RA in the Iranian population. The findings suggest that the CB2 receptor—specifically the Q63R variant—could serve as a potential diagnostic biomarker for identifying individuals at higher risk of developing RA. Therefore, further large-scale studies are needed to elucidate the role of the CB2 receptor in RA susceptibility, as well as its diagnostic and therapeutic potential in clinical applications.

## Figures and Tables

**Table 1:** Demographic, clinical, and treatment characteristics of subjects (*n* = 240).

	**All subjects**	**Patients**	**Controls**
*Sex*			
Male, *n* (%)	43 (17.9)	23 (19.2)	20 (16.7)
Female, *n* (%)	197 (82.1)	97 (80.8)	100 (83.3)
Sex ratio (male: female)	1:4.5	1:4.2	1:5
Age (year)			
Median	50.5	51	50
Mean	50.2 ± 12.6	50.1 ± 13.8	50.3 ± 11.4

*Job* ^a^			
Housework, *n* (%)	81 (33.75)	52 (43.3)	29 (24.16)
Retired, *n* (%)	27 (11.25)	5 (4.2)	22 (18.33)
Self-employment, *n* (%)	21 (8.75)	4 (3.3)	17 (14.16)
Other jobs, *n* (%)	67 (27.91)	19 (15.8)	48 (40)

*RF test* ^a^			
RF positive, *n* (%)	78 (32.5)	78 (65)	—
RF negative, *n* (%)	150 (62.5)	30 (25)	—
Anti-CCP test^a^			
Anti-CCP positive, *n* (%)	68 (28.3)	68 (56.7)	—
Anti-CCP negative, *n* (%)	157 (65.4)	37 (30.8)	—

*Treatment*			
DMARD, *n* (%)	56 (23.3)	56 (46.6)	—
Steroid, *n* (%)	52 (21.65)	52 (43.3)	—
NSAID, *n* (%)	12 (5)	12 (10)	—
Azathioprine, *n* (%)	9 (3.75)	9 (7.5)	—
Biologic (Adalimumab), *n* (%)	2 (0.8)	2 (1.6)	—

Abbreviations: Anti-CCP, anticyclic citrullinated peptide; DMARD, disease-modifying antirheumatic drugs; NSAID, nonsteroidal anti-inflammatory drugs; RF, rheumatoid factor.

^a^Missing in variables of job, RF test, and anti-CCP test were 18.3%, 5% and 6.3%, respectively.

**Table 2:** Allele and genotype frequencies of Q63R polymorphism in subjects (*n* = 240).

**Allele**	**All subjects**		**Patients**		**Controls**	
	**Count**	**Proportion**	**Count**	**Proportion**	**Count**	**Proportion**
R	276	0.575	151	0.63	125	0.52
Q	204	0.425	89	0.37	115	0.48

*Genotype*						
RR	72	0.30	45	0.38	27	0.225
QR	132	0.55	61	0.51	71	0.591
QQ	36	0.15	14	0.12	22	0.183

**Table 3:** Demographic and clinical characteristics of subjects according to Q63R polymorphism (*n* = 240).

	**RR**	**QR**	**QQ**	**Total**	**p** ** value**
*Sex*					0.75
Male, *n* (%)	12 (16.7)	23 (17.4)	8 (22.2)	43 (17.9)	
Female, *n* (%)	60 (83.3)	109 (82.6)	28 (77.8)	197 (82.1)	
Total, *n* (%)	72 (100)	132 (100)	36 (100)	240 (100)	

*Job* ^a^					0.58
Housework, *n* (%)	26 (48.1)	44 (41.1)	11 (31.4)	81 (41.3)	
Retired, *n* (%)	7 (13)	12 (11.2)	8 (22.9)	27 (13.8)	
Self-employment, *n* (%)	5 (9.3)	12 (11.2)	4 (11.4)	21 (10.7)	
Other jobs, *n* (%)	16 (29.6)	39 (36.4)	12 (34.3)	67 (34.2)	
Total, *n* (%)	54 (100)	107 (100)	35 (100)	196 (100)	

*RF test* ^a^					0.15
RF positive, *n* (%)	30 (42.9)	39 (31.7)	9 (25.7)	78 (34.2)	
RF negative, *n* (%)	40 (57.1)	84 (68.3)	26 (74.3)	150 (65.8)	
Total, *n* (%)	70 (100)	123 (100)	35 (100)	228 (100)	

*Anti-CCP test* ^a^					0.16
Anti-CCP positive, *n* (%)	26 (37.7)	35 (28.9)	7 (20)	68 (30.2)	
Anti-CCP negative, *n* (%)	43 (62.3)	86 (71.1)	28 (80)	157 (69.8)	
Total, *n* (%)	69 (100)	121 (100)	35 (100)	225 (100)	

Abbreviations: Anti-CCP, anti-cyclic citrullinated peptide; RF, rheumatoid factor.

^a^Missing in variables of job, RF test, and anti-CCP test were 18.3%, 5%, and 6.3%, respectively.

**Table 4:** Association of Q63R polymorphism with rheumatoid arthritis (*n* = 240).

**Model**	**Genotype**	**Patients (%)**	**Controls (%)**	**OR (95% CI)**	**p** ** value**	**AIC**	**OR (95% CI)**	**p** ** value**	**AIC**
				**Not adjusted**			**Adjusted by covariates** ^**a**^		
Codominant	RR	45 (37.5)	27 (22.5)	1.00	0.029	331.6	1.00	0.027	335.2
	QR	61 (50.8)	71 (59.2)	1.94 (1.08–3.49)			1.94 (1.08–3.50)		
	QQ	14 (11.7)	22 (18.3)	2.62 (1.15–5.96)			2.65 (1.16–6.07)		

Dominant	RR	45 (37.5)	27 (22.5)	1.00	0.011	330.2	1.00	0.01	333.9
	QR-QQ	75 (62.5)	93 (77.5)	2.07 (1.17–3.64)			2.08 (1.18–3.66)		

Recessive	RR-QR	106 (88.3)	98 (81.7)	1.00	0.15	334.6	1.00	0.14	338.3
	QQ	14 (11.7)	22 (18.3)	1.70 (0.82–3.51)			1.72 (0.83–3.57)		

Overdominant	RR-QQ	59 (49.2)	49 (40.8)	1.00	0.19	335	1.00	0.19	338.8
	QR	61 (50.8)	71 (59.2)	1.40 (0.84–2.33)			1.40 (0.84–2.34)		

Log-additive	—	—	—	1.68 (1.13–2.50)	0.0099	330.1	1.69 (1.13–2.52)	0.009	333.7

Abbreviations: 95% CI, 95% confidence intervals; AIC, Akaike information criterion; OR, odds ratio.

^a^Covariate is age and gender∗.

## Data Availability

The authors confirm that the data supporting the findings of this study are available within the article.

## Nomenclature

RA: rheumatoid arthritis
RF: rheumatoid factor
PPP: purchasing power parity
PTPN22: tyrosine phosphatases nonreceptor Type 22
HLA-DR: human leukocyte Antigen D-related
TRAF1/C5: tumors necrosis factor–receptor associated Factor 1 and complement Component 5
IRF-5: regulatory Factor 5
ECS: endocannabinoid system
CB1: cannabinoid Receptor 1
CB2: cannabinoid Receptor 2
GPCR: G-protein-coupled receptor
*CNR2*: cannabinoid CB2 receptor gene
ITP: childhood immune thrombocytopenic purpura
JIA: oligo/polyarticular juvenile idiopathic arthritis
MS: multiple sclerosis
ORs: odds ratios
CIs: confidence intervals
IBD: inflammatory bowel disease
HCV: Hepatitis C virus
HIV: human immunodeficiency virus
